# Case Characterization, Clinical Features and Risk Factors in Drug-Induced Liver Injury

**DOI:** 10.3390/ijms17050714

**Published:** 2016-05-12

**Authors:** Aida Ortega-Alonso, Camilla Stephens, M. Isabel Lucena, Raúl J. Andrade

**Affiliations:** 1 Unidad de Gestión Clínica de Enfermedades Digestivas y Farmacología Clínica, Instituto de Investigación Biomédica de Málaga (IBIMA), Hospital Universitario Virgen de la Victoria, Universidad de Málaga, 29071 Málaga, Spain; aida_ortega_alonso@hotmail.com (A.O.-A.); cstephens@uma.es (C.S.); lucena@uma.es (M.I.L.); 2 Centro de Investigación Biomédica en Red de Enfermedades Hepáticas y Digestivas (CIBERehd), 28029 Madrid, Spain

**Keywords:** drug-induced liver injury, DILI, risk factors

## Abstract

Idiosyncratic drug-induced liver injury (DILI) caused by xenobiotics (drugs, herbals and dietary supplements) presents with a range of both phenotypes and severity, from acute hepatitis indistinguishable of viral hepatitis to autoimmune syndromes, steatosis or rare chronic vascular syndromes, and from asymptomatic liver test abnormalities to acute liver failure. DILI pathogenesis is complex, depending on the interaction of drug physicochemical properties and host factors. The awareness of risk factors for DILI is arising from the analysis of large databases of DILI cases included in Registries and Consortia networks around the world. These networks are also enabling in-depth phenotyping with the identification of predictors for severe outcome, including acute liver failure and mortality/liver transplantation. Genome wide association studies taking advantage of these large cohorts have identified several alleles from the major histocompatibility complex system indicating a fundamental role of the adaptive immune system in DILI pathogenesis. Correct case definition and characterization is crucial for appropriate phenotyping, which in turn will strengthen sample collection for genotypic and future biomarkers studies.

## 1. Introduction

Drug-induced liver injury (DILI) is probably one of the most intriguing and complex liver diseases because unlike other safety problems with the use of drugs, the facets of hepatotoxicity are multiple, difficult to approach and with great potential impact in clinical drug development. DILI is a broad condition that symptomatically can mimic most kinds of acute and chronic liver conditions. Despite stringent requirements for drug development imposed by regulatory agencies, DILI is an increasing health problem and a significant cause for failure to approve drugs, market withdrawal of commercialized medications and adoption of regulatory measures. Toxic liver disease is a challenging differential diagnosis for the doctors, not only because of its potential severity, but also by the inability to establish a definitive diagnosis in most cases. Acute DILI has around a 10% probability of evolving into chronic and severe forms, and even be fulminant [1,2]. Gastroenterologists should always consider DILI in all patients with unexplained acute or chronic liver damage. The causal agents of this condition were initially restricted to pharmaceutical products, but it is now apparent that many herbal and dietary supplements (HDS) can also produce hepatotoxicity [3].

Data on DILI incidence in the general population are very limited. In a prospective study of a population of 80,000 inhabitants of northern France, conducted during 1997–2000, the incidence of DILI was 13.9 cases per 100,000 patients-year, a rate 16 times higher than those reported to the French National Agency [4]. More recently a population-based study in Iceland [5], reported an incidence of DILI of 19.1 cases per 100.000 inhabitants-year. Is important to highlight that in the Iceland study, liver injury by acetaminophen was excluded; and, in the French study, HDS were not mentioned. Efforts to enhance the identification of adverse hepatic reactions and to obtain reliable information on DILI epidemiology and pathogenesis have been made with the development of large DILI Registries. The Spanish DILI Registry (www.spanishdili.uma.es) and the Drug-Induced Liver Injury Network (http://dilin.duke.edu) are collaborative multicenter networks with large databases and biosample collections of prospectively recorded DILI cases in Spain and the US, respectively, and subsequently important resources for hepatotoxicity studies. In population based studies there are a greater proportion of asymptomatic cases in comparison with the cases included in the DILI Registries probably because of the selection bias of reporting to Registries only the more severe cases [2,6]. The incidence rate of idiosyncratic DILI is generally believed to be in the range of 1–10 in every 10.000 exposed individual, with some medications known to cause DILI more frequently than others. The more common pharmacological groups responsible for hepatotoxicity in Western countries include anti-infective, anti-inflammatory and nervous system drugs, with amoxicillin-clavulanate being the most causative single agent [2,6], while herbal medications are still a major component of hepatotoxicity in Eastern countries [7].

In this review, we addressed aspects on phenotype characterization, clinical presentation and outcomes, as well as the main risk factors both from the drug and the host known to be involved in idiosyncratic drug induced liver injury.

## 2. Case Characterization

DILI has a very broad spectrum of presentation, both in phenotype and severity this last ranging from asymptomatic elevations of liver aminotransferases to acute liver failure. DILI can mimic almost every other liver disorder including rare syndromes such as vascular disorders and liver tumors although the most common presentation is an acute viral hepatitis-like syndrome [8]. Despite its rarity DILI has been reported as the most commonly implicated reason for acute liver failure (ALF) in the US, with acetaminophen overdose being responsible for 39% and idiosyncratic DILI 13% with antibiotics being the main causative drug group, of ALF cases [9,10]. Symptoms are quite unspecific. In the U.S. Drug Induced Liver Injury Network (DILIN) registry of over 1200 consecutive cases, nausea was present in 60% and abdominal pain in 42% [11]. The lack of reliable and sensitive biomarkers that can distinguish DILI from other causes of liver injury is indeed a main difficulty facing physicians in real practice.

In the clinical setting, case characterization usually does not rely on liver histopathological manifestations because liver biopsy is not routinely performed in suspected cases of hepatotoxicity. Instead, liver biochemistry is used to define liver injury. The analytical determinants for DILI has changed over the last 20 years and today discrete elevations of aminotransferases have an uncertain meaning as modest elevations in the liver profile is now more frequently seen possibly due to the growing prevalence of non-alcoholic fatty liver disease in general population and the fact that some drugs (*i.e.*, statins) can occasionally induce minor and transient alterations in liver tests. Hence, a consensus group proposed to meet one of the following criteria as a prerequisite to consider a case like DILI [12]: (a) alanin-aminotransferase (ALT) value ≥5 times of upper limit of normal (ULN); (b) alkaline phosphatase (ALP) value ≥2 × ULN or (c) ALT value ≥3 × ULN and total bilirubin (TB) ≥2 × ULN.

Accordingly, the phenotypes of liver injury are also defined by biochemical criteria [12]. The pattern of damage is defined using the value of R, where *R* = (ALT patient/ULN)/(ALP patient/ULN). The resultant pattern is classified as hepatocellular (*R* ≥ 5), cholestatic (*R* ≤ 2) and mixed cases (*R* > 2 and <5) (Table 1).

The international consensus also recommended that AST substitute ALT when the latter is unavailable [12]. On the other hand, γ-glutamil transpetidase (GGT) is sometimes used as a surrogates of ALP but its reliability as biomarker of cholestasis in DILI is unclear. A recent analysis from the Spanish DILI Registry cohort showed that AST could reliably replace ALT when calculating pattern of liver injury in DILI, while GGT can only substitute ALP when the resultant *R*-value scores as hepatocellular [13]. The first available blood test after DILI initiation should be used to determine liver injury type as the pattern of liver injury could change over time, predominantly with a transition towards a lower *R*-value during disease progression [1]. In the last analysis of DILIN [6] the pattern of liver injury was hepatocellular in 54%, and cholestatic or mixed in 23% each; these results are similar to those previously published by the Spanish DILI Registry [2] (58% hepatocellular, 20% cholestatic and 22% mixed) indicating that the use of liver biochemistry for DILI case characterization leads to good agreement between non-homogenous DILI populations. Very recently the Spanish DILI Group proposed the calculation of a new R (*nR*), (ALT or aspartate-aminotransferase (AST), whichever was highest, /ULN/(ALP/ULN) at DILI onset. The rationale for assessing this *nR* was based on the finding that AST level was independently associated with the development of acute liver failure/orthotopic liver transplantation (OLT) at all-time points being most predictive at DILI recognition [14].

There is no standardized histological system to classify DILI. The DILIN group has reported the pathological findings in over 249 DILI cases, in which 18 histopathological patterns were predefined, although 83% of the cases could be classified into one of five patterns: acute hepatitis; chronic hepatitis; acute cholestasis; chronic cholestasis; and cholestatic hepatitis. Interestingly, the correlation between liver histology and biochemistry was fair as there were an important overlap among the different patterns with regard to the *R* value [15].

Nevertheless, tentative correlations between histological findings and biochemical classifications found lobular disarray and rosette formation to be more prevalent in hepatocellular cases (*R* ≥ 5), while bile plugs and duct paucity appeared more commonly in cholestatic cases (*R* ≤ 2) [15]. However, liver biopsy is not a routine procedure in DILI assessment, and in cases where a biopsy is performed it may be delayed with DILI onset.

The degree of elevation of enzyme levels alone may not reflect the severity of liver injury because these values do not accurately predict specific clinical outcomes. Hence, a consensus group graded severity taking into consideration clinical and laboratory features [12] as follows: (a) Mild: Elevated ALT or ALP values reaching criteria for DILI, but TB < 2 × ULN; (b) Moderate*:* Elevated ALT/ALP values reaching criteria for DILI and TB ≥ 2 × ULN, or symptomatic hepatitis; (c) Severe*:* Elevated ALT/ALP values reaching criteria for DILI, T ≥ 2 × ULN, and one of the following: (1) International normalized ratio (INR) ≥1.5; (2) ascites and/or encephalopathy, disease duration <26 weeks, and absence of underlying cirrhosis or (3) other organ failure considered to be due to DILI; and (d) Fatal: Death or transplantation due to DILI. Similarly, the DILIN group developed an operational procedure system for severity classification [16]. The distribution of the cases in the DILIN cohort [6] in their most recent update and according to these criteria is: 24% mild, 21% moderate, 29% moderate (with hospitalization), 19% severe and 7% fatal (Table 2).

With regard to the prediction of severe outcomes in DILI, in the late 1960s it was recognized by Zimmerman, the pioneer of modern liver toxicology, that a patient who presents with jaundice as a result of a drug-induced hepatocellular injury (without a significant obstructive component and once other causes for increased bilirubin levels such as hemolysis or Gilbert syndrome are excluded) had at least a 10% (and, in some instances, as many as 50%) chance of fatal liver failure [17]. This is because the rise in TB as a result of drug-induced hepatocellular injury reflects a major loss of functioning hepatocytes. Zimmerman’s observation has been referred to by Robert Temple (a former FDA director) as “Hy’s Law”. The Hy’s law was prospectively validated in the Spanish DILI cohort that showed a mortality/liver transplantation rate of 11.7% in DILI patients with hepatocellular jaundice [14]. However, the classical definition of Hy’s Law is quite sensitive but lack specificity since encompasses not only hepatocellular but also cholestatic and mixed cases that evolve less frequently to acute liver failure. Interestingly, the FDA guidance for clinical trials [18] described as criterion for fulfilling “Hy’s Law” that in addition to the hepatocellular nature of the liver injury “there should not be a prominent cholestatic component”. Hence, the best definition of Hy’s Law cases has been a matter of debate.

Recently, the Spanish DILI Group [14] has reassessed the role of elevated levels of alkaline phosphatase (>2 × ULN) in DILI patients who otherwise met Hy’s law criteria showing that there were no difference in outcome in this subset of patients as compared with those with lower ALP levels. Hence, the authors proposed a redefinition of the “Hy’s Law” that encompasses all subjects with drug-induced hepatocellular injury (*R* or *nR* ≥ 5) accompanied by hyperbilirubinemia, without excluding cases based on ALP level. This group also developed a composite algorithm to predict an ALF outcome in idiosyncratic DILI cases which showed improved specificity (82%) and sensitivity (80%). This algorithm, which still requires further validation in a larger prospective DILI cohort, is based on analytical parameters (serum total bilirubin, AST elevation and AST/ALT ratio) from the first available blood test after DILI initiation [14] and is intended to be used by physician in clinical practice for early evaluation of DILI cases in order to provide appropriate care. Histologic findings have been also found to show some prognostic significance in Kleiner *et al.* [15] study; necrosis, fibrosis, and microvesicular steatosis being associated with worse outcomes, whereas granulomas and eosinophilic infiltrates were associated with better outcomes [15]. Interestingly, Spanish DILI patients exhibiting a low or intermediate interleukin (IL)-10 producing haplotype, leading to lower eosinophil counts, showed more severe DILI outcome [19]. This finding also raises the possibility that patients with low levels of IL-10, which is an immunoregulatory citokyne involved in immune tolerance, have a defective clinical adaptation.

## 3. Clinical Features

The most common form of presentation of DILI is an acute viral “hepatitis-like” syndrome, with jaundice, nausea, fatigue and abdominal discomfort or pain [20]. However, DILI can virtually mimic any other liver disease and phenotype such as acute cholestasis, chronic hepatitis, or more rarely cirrhosis, veno-occlusive disease and even neoplasms [21]. Table 3, shows the expression of hepatotoxicity with many commonly used drugs. For many drugs a signature pattern in both latency and clinical expression is proposed but it must be kept in mind that a drug signature varies and, hence, is of limited value for diagnostic purposes (Table 4) [15,22,23,24].

Acute hepatocellular injury is defined as ALT ≥ 5 × ULN or *R* ≥ 5. Sometimes there are features of hypersensitivity, such as fever, rash or peripheral eosinophilia, suggesting drug allergy. This phenotype is more frequent in young females (65% females with mean age of 45 years) in the DILIN analysis [6]. Histologically, can be found varying degrees of inflammation and necrosis can be found, but the centrilobular prevalence of lesions and the presence of inflammatory infiltrate rich in eosinophils suggest a toxic etiology [25,26]. The centrilobular necrosis is particularly prominent in cases of poisoning with some intrinsic hepatotoxins such as paracetamol, or cocaine [8]. In hepatocellular damage there is a higher risk of ALF, as Zimmerman announced in his “Hy’s Law” and we have discussed above. The last published update of DILIN database [6] described a higher frequency of fatal cases (liver-related death or transplantation) in the hepatocellular cases (9% cases) than in cholestatic or mixed cases (4% in each), with 6.2% of OLT in the first group. These observations are similar to those published data by the Spanish DILI Group in 2005 [2]: (6% ALF and 3% OLT in hepatocellular group *versus* 1% ALF/OLT in cholestatic group).

Acute cholestatic injury is defined as ALP > 2 × ULN or *R* < 2, and there are two subtypes: pure cholestasis (bland or canalicular) and acute cholestasis or hepatocanalicular hepatitis. The probability of a cholestatic phenotype increases with advancing age and is more common in males [27]. Its usual presentation is with jaundice and itching [8].

Pure cholestasis is characterized by increased serum levels of conjugated bilirubin, ALP and G-glutamyl transpeptidase (GGT) with little or no alteration of transaminases. Histologically, it presents with hepatocyte cholestasis and dilated biliary canaliculi with bile plugs, without evidence of necrosis or inflammation. This type of lesion is characteristic of contraceptives and anabolic steroids [28].

In Hepatocanalicular hepatitis there may be fever and abdominal pain simulating acute biliary obstruction, and often associated hypersensitivity features. Pathological findings include, portal and ductal inflammation as well as hepatocyte necrosis with marked predominance centrilobular cholestasis predominance [25].

Among the drugs involved in this subtype are amoxicillin-clavulanate, macrolides, azithromycin [29], fluoroquinolones [30] and phenothiazines. In some cases the lesion can progress to “vanishing bile duct syndrome”, with persistent cholestasis and even biliary cirrhosis may develop, being chlorpromazine the prototype drug that can lead to this rare outcome [31].

Mixed hepatic injury is defined as R between 2 and 5 being clinical and laboratory abnormalities intermediate between the cholestatic and hepatocellular injury. The manifestations of drug allergy are more common. Almost all drugs that induce cholestatic hepatitis can also cause this type of liver injury [8].

Chronic DILI (unresolved injury 6 months after onset) was much more frequent among cholestatic cases (31%) than either hepatocellular (13%) or mixed-injury cases (14%) in the DILIN analysis [6]. In the Spanish Registry, the results were similar: cholestatic/mixed type of damage (18 of 194 cases (9%)) was more prone to chronic outcome than hepatocellular injury (10 of 240 cases (4%)) [1].

The definition of chronicity in DILI remains a matter of debate, The International Consensus Conference [32] it is recommended to define a case as chronic when the alteration persists more than 3 months in the hepatocellular type of damage and more than 6 months in the cholestatic and mixed ones. However, a recent prospective natural history study indicates that up to 8% of cases persist with laboratory abnormality during the first year of follow-up [33]. This time-point cutoff is considered the most suitable for establishing chronicity as the bulk of patients who normalized liver biochemistry did so within one year after stopping the offending drug, regardless of their phenotype of injury [12]. Furthermore, elevations in ALP > 1.1 × ULN and TB > 2.8 × ULN in the second month after DILI onset could be predictive of chronicity in hepatotoxicity [33]. These findings bear relevant clinical implications in the monitoring of patients after an acute DILI episode.

An increasingly reported phenotype is that of DILI with autoimmune features [34]. This phenotype ranges from DILI patients with positive autoantibodies (antinuclear antibodies, antismooth muscle antibodies and anti-LKM antibodies) without accompanying specific features, up to a picture indistinguishable from “idiopathic” autoimmune hepatitis (AIH), with high serum IgG levels and interface hepatitis in liver biopsy, the so called drug-induced-AIH [35]. This last phenotype is hard to be linked to a particular drug since there are no specific histological or genetic biomarkers that can confidently establish the diagnosis so the drug could be an “innocent” bystander or indeed unmask an autoimmune hepatitis in a predisposed subject. Moreover, because the process may self-perpetuate upon drug-discontinuation causality assessment is specially challenging yielding low scores when applying by the RUCAM scale [36]. Characteristics than can point towards a the diagnosis of drug-induced AIH are the absence of cirrhosis in liver biopsy and the long-term maintenance of remission once steroids are tapered [37]. Portal infiltrating B cells (CD20^+^) were more numerous in immunostains of liver biopsies from patients with idiopathic AIH as compared with those of drug-induced AIH [38]. The latency period is variable but can be prolonged, in some cases even for several years after starting the drug. The compounds which have been associated to drug-induced AIH include herbal products and drugs such as methyldopa, minocycline, nitrofurantoin, diclofenac [34], and more recently biological agents [39] and statins [40].

Recently, immune check-point blockade by manipulating either cytotoxic-T lymphocyte A-4 (CTLA-4) or programed death-1 (PD-1) receptors has attracted attention in its relationship to idiosyncratic DILI pathogenesis indirectly supporting the importance of immune pathways. CTLA-4 mediated immune checkpoint is induced in T cells at the time of their initial response to antigen, dampening the amplitude of the initial response. In contrast, the major role of the PD1—an inhibitory molecule of lymphocyte activation-pathway is not at the initial T cell activation stage but rather to regulate inflammatory responses in tissues by effector T cells recognizing antigen in peripheral tissues. Thus, immune checkpoint blockade leads to vigorous immune responses and this concept is used to uncover potent antitumor immune responses that are depressed in cancer [41]. Interestingly, CTLA-4 immunotherapeutic agents used in advanced cancer such as the anti-CTLA-4 monoclonal antibody ipilimumab, which augments T-cell activation and proliferation, have been associated with immune-mediated multi-organ damage, including several cases of hepatitis and liver failure [42].

Steatosis is another clinicopathological pattern that can result from the toxic effect of drugs. Macrovesicular steatosis is characterized by variable degrees of accumulation of large fat droplets with peripheral displacement of the nucleus without significant inflammation or cholestasis. Tetracyclines, steroids, gold, 5-fluorouracil, methotrexate or tamoxifen are examples of drugs capable of inducing this pattern of injury [22,23]. The microvesicular type is characterized by diffuse hepatocyte accumulation of small fat droplets maintaining a central placement of the nucleus without significant inflammation or cholestasis or alternate pattern. This pattern is related to valproic acid, tetracyclines or zidovudine [22,23]. Steatosis is frequently present in liver biopsies of DILI patients in association with other patterns—65% of the cases series of Kleiner *et al*. [15] (73% macrovesicular, 14% mixed and 13%, microvesicular).

Sinusoidal obstruction syndrome (SOS; veno-occlusive disease) is secondary to endothelial cell injury to small hepatic venules that manifests as endothelial swelling and thrombosis. This pattern is related to cytotoxic drugs, myeloablation before stem cell transplantation and bone marrow transplantation [23]. Genetic polymorphisms in methylenetetrahydrofolate reductase have been implicated in SOS in post-transplant patients [43]. Some other DILI cases that do not exhibit autoimmune features present with associated extrahepatic manifestations such as skin lesions (rash, Stevens-Johnson or Lyell syndrome), fever, hematological manifestations (eosinophilia, granulocytopenia, thrombocytopenia or hemolytic anemia) and involvement of other organs (kidney, pancreas) all of which are strongly suggestive of drug hypersensitivity (immune-mediated) favoring the diagnosis of DILI. However, these signs occur in a minority of cases of hepatotoxicity, (23% of the cases in the Spanish cohort [2]) so their sensitivity and positive predictive value is low. The DILIN reported 9 out of 899 subjects with severe cutaneous adverse reactions (Stevens Johnson syndrome and toxic epidermal necrolysis) [6]. It is important to underscore that the manifestations of allergy do not appear consistently, even with the same agent.

## 4. Risk Factors

The idiosyncratic DILI is basically thought to be due to the interaction of three factors: a drug that has the potential to be harmful to the liver, a genetically susceptible subject and the intervention of other host and environmental factors [34]. The rarity of idiosyncratic liver damage upon exposure to otherwise safe drugs for the bulk of subjects has suggested that it mostly rely on genetic variants that make the subject susceptible. Genetic studies, either with a design of candidate genes involvement in drug metabolism (Phase I–III) or without a priori assumptions (Genome-wide association study or GWAs) have found significant associations in cases of DILI [44]. GWAS conducted in groups of cases of DILI caused by specific drugs such as flucloxacillin, amoxicillin-clavulanate, ximelagatran, lapatinib or lumiracoxib, have consistently identified signals in the “Manhattan plot” corresponding to the HLA (Human Leukocyte Antigen) region of chromosome 6 both class I and class II [45,46,47,48,49,50] underscoring the fundamental (and probably restrictive in most instances) role of the adaptive immune system in DILI susceptibility. Because of the enrichment of DILI networks with cases of amoxicillin-clavulanate and flucoxacillin made difficult to detect other signals a recent analysis involving 878 DILI cases related to multiple drugs, in which flucloxacillin and co-amoxiclav were excluded reported a novel genome-wide significant association of HLA-A*33:01 with all causes of DILI and strongly with terbinafine, fenofibrate and ticlopidine [50] (Table 5).

Therefore, only in carriers of the HLA risk alleles would occurs the presentation of the haptenized reactive metabolite by antigen-presenting cells to the adaptive immune system (“hapten hypothesis”) [51,52].

**Figure 1:**
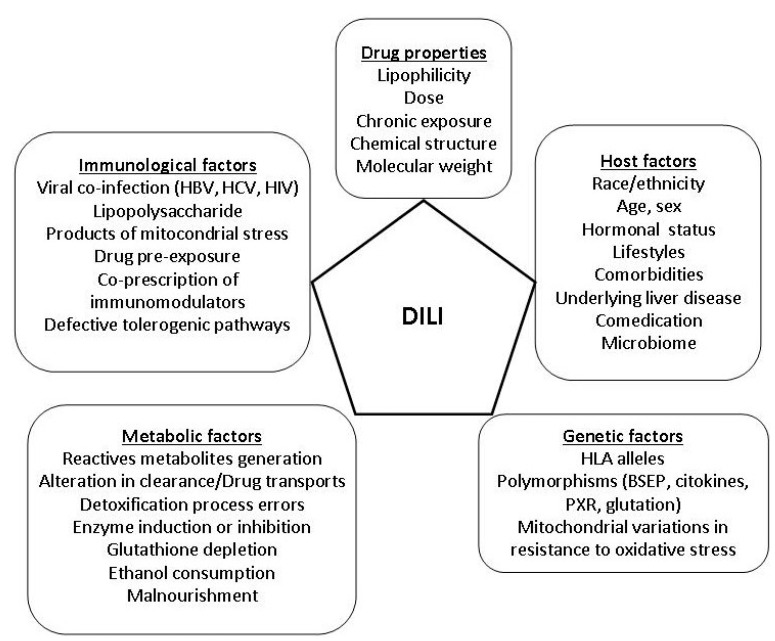
Risk factors in DILI.

Often, it is necessary a co-stimulus initiated by releasing DAMPS (Damage Associated Molecular Patterns), which are released by the reactive metabolites that damage liver cells and starts a sterile inflammation, which stimulates the innate immune system through the “toll-like receptors” of antigen-presenting cells. The production of proinflammatory cytokines that follows stimulate the adaptive immune system, which induces a direct cytotoxic response or mediated by antibodies against hepatocytes (“danger signal hyphotesis”). For many drugs in which the risk of DILI is associated with certain HLA alleles, toxicity will occur only in carriers of the alleles of interest, but people without this genetic profile would be free of risk [53]. In contrast, only a minority of subjects with risk alleles exposed to the drug develop DILI, because other drug and host factors are necessary companions. (Figure 1) [54].

### 4.1. Drug Factors

#### 4.1.1. Drug Dose and Lipophilicity

DILI is generally divided into intrinsic and idiosyncratic reactions based on its dose dependency and predictability. Intrinsic hepatotoxicity is dose dependent and can be predicted in animal models. Idiosyncratic DILI is not related to the pharmacological properties of a drug and, hence, unpredictable. However, it is now becoming apparent that idiosyncratic DILI is not entirely a dose independent event. In fact, drugs given at a low daily dose are rarely associated with a high incidence of idiosyncratic ADRs, because even for allergic reactions a threshold dose (≥10 mg) is usually required [55]. Furthermore, a study of pharmacy databases has demonstrated an association of serious hepatic events such as liver failure, liver transplantation and liver-related death with higher dosage drugs (≥50 mg per day) [56]. This finding has been corroborated by data from large cohorts in Iceland and Spain, in which drugs with a recommended daily dose of ≥50 mg were responsible for 88% and 77%, respectively, of all DILI cases [5,27]. Likewise, drugs that undergo extensive hepatic metabolism (>50%) are also associated with a greater risk of idiosyncratic hepatotoxicity [57]. Indeed, it has been argued that surpassing a threshold dose in a given susceptible individual may favor DILI occurrence [58].

Lipophilicity is an important pharmacological property, correlating with drug uptake and metabolism. Analysis of large sets of compounds revealed that the lipophilic compounds are more likely to produce toxic events [59,60].

The combination of both factors in a given drug (daily dose > 100 mg/day) and high lipophilicity (calculated octanol-water partition coefficient (log*P* > 3), which is indeed, simply a surrogate for extensive biotransformation and hepatic exposure to a parent drug or reactive metabolite has been called the “rule of 2” and was present among a majority of drugs with proven liver toxicity in two independent databases of the Food and Drug Administration (FDA)-approved drugs labeled for the presence or absence of liver injury [61]. In contrast, Weng *et al*. [62] did not find a sinergisitic effect of the combination of defined daily dose and lipophilicity in predicting hepatic adverse events compared to using defined daily dose alone. In this last study hepatic metabolism of the drug but not lipophilicity was actually predictive of the risk of hepatotoxicity.

#### 4.1.2. Reactive Metabolites and Oxidative Stress

Many drugs undergo biotransformation via Phase I metabolic pathways leading to the formation of metabolites that differ from the parent drug in terms of chemical activity. These metabolites can covalently bind to proteins to create a complex drug-protein complex and produce either direct toxicity or mediated by the immune system [52]. The important role of reactive metabolites in the pathogenesis of idiosyncratic DILI is a long-term assumption yet lacking a body of evidence. There is no clear correlation between the potential to form reactive metabolites *in vitro* and incidence of hepatotoxicity [63], and its formation is not a prerequisite for DILI development. For example, pemoline, which has been withdrawn due to idiosyncratic toxicity, does not appear to produce reactive metabolites [64].

Obach *et al.* [65] analyzing covalent binding of drugs in human liver microsomes *in vitro* could not distinguish between hepatotoxic and non-hepatotoxic compounds. Bauman *et al*. [66] found similar results when used human hepatocytes or macromolecules in human liver S-9 fractions, showing that data of covalent binding *in vitro* hardly predicts idiosyncratic hepatotoxicity.

Reactive drug metabolite formation can also lead to oxidative stress a disturbance in the balance between cellular pro- and anti-oxidant activities. Increased reactive oxygen species (ROS) can directly damage DNA, proteins, enzymes, and lipids in cells and tissues and induce immune-mediated liver damage [52]. In an *in vitro* approach, Xu *et al*. [67] used content cellular imaging in primary human hepatocyte cultures that when applied to over 300 drugs and chemicals including well-known hepatotoxic compounds in humans identified ROS generation, mitochondrial damage and intracellular gluthatione depletion as the most important factors contributing to the hepatotoxicity.

In a recent study [68] in rat livers to identify drug compounds that induce cellular oxidative stress, various drugs (carbamazepine, chlorpromazine, clozapine) were associated with a specific expression signature. Other drugs (ex. linezolid and tacrine) did not generated the determined expression signature.

#### 4.1.3. Mitochondrial Hazards

The crucial role of mitochondria as cellular energy supplier can explain its importance in the pathogenesis of DILI. Mitochondrial damage can trigger apoptosis and/or hepatic necrosis, leading to the activation of a signaling pathway of cell death when a threshold of mitochondrial damage is exceeded [52]. It has been estimated that this threshold for cellular phenotype changes due to large scale mtDNA deletions or tRNA point mutations exceeds 60% and 90%, respectively [69]. Experimentally, many drugs related to DILI have shown to be harmful to mitochondria [70,71]. Recent studies show that some drugs can damage specific mitochondrial processes: diclofenac (membrane disruption), tacrine (mtDNA), valproic acid (respiration), tamoxifen and aspirin (β-oxidation) [72,73,74]. However, the main mitochondrial mechanism that is the primary target of a specific drug is unknown. Electron transfer impairment in the respiratory chain will have a negative effect on fatty acid oxidation and will increase ROS production, that may cause mtDNA damage. On the other hand, drugs can interfere with mtDNA replication or gene expression and harm the electron transfer process (inhibit or reduce the production of protein complexes of the respiratory chain).

A recent study [71] on isolated mouse liver mitochondria analyzed 124 compounds and reported highly significant relationship between drug-induced mitochondrial toxicity and DILI occurrence. However, drug concentrations used in experimental studies are often significantly higher than those attained in patients so the results of these studies should be interpreted with caution. Patients homozygous to common genetic variants of the manganese superoxide dismutase (*SOD2 Ala*) and glutathione peroxidase (*GPX1 Leu*) genes, which are involved in mitochondrial oxidative stress, are more prone to develop cholestatic DILI particularly from drugs forming electrophilic metabolites or that are mitochondrial hazardous [75].

An interesting hypothesis gives support to the antibiotics risk of inducing mitochondrial impairment. Being of bacterial ancestry, the mitochondria share many genetic and structural similarities with bacteria that could render mitochondria more vulnerable towards antibiotics. This may explain, at least in part, (other explanation being their effects on microbioma) why antibiotics top the list of causative agents among DILI cases identified to date [76].

#### 4.1.4. Hepatobiliary Transporter Inhibition

Inhibition of the hepatic bile salts efflux transporter bile salt export pump (BSEP) leads to accumulation of toxic bile salts in hepatocytes, which could lead to cell damage. Consequently, drugs that exhibit inhibitory effects on BSEP can have hepatotoxic potential [77]. *In vitro* testing of 200 benchmark compounds by the use of human BSEP inverted vesicles demonstrated that most tested compounds found to have potent inhibitory effect on BSEP (IC_50_ ≤ 25 µM) for example troglitazone, ketoconazole, nefazodone and lapatinib, have been associated with human liver liabilities [78]. In another *in vitro* testing the median potency of BSEP inhibition was higher among drugs that caused cholestatic/mixed DILI than among drugs that caused hepatocellular or no DILI [79]. However, there are many false negative with this approach as many drugs implicated in hepatotoxicity are weak inhibitors of BSEP [78]. Interestingly, however, conditions of oxidative stress can internalize transporters such as BSEP and subsequently impair bile salt secretory function [80]. Thus, drugs without capacity to significantly inhibit BSEP directly may still affect transporter activity indirectly through induction of oxidative stress. Furthermore, as BSEP is an ATP-dependent transporter, it has been hypothesized that drugs being potent inhibitors of both BSEP activity and mitochondrial function would induce more frequently severe hepatotoxicity. In a comparison of 72 drugs, Aleo *et al*. [81] found that drugs with dual effects on mitochondrial and BSEP inhibition were associated with more severe DILI than another with only one of them.

However, *in vitro* models focusing on a single transporter do not reflect the true activity *in vivo.* In cholestatic conditions, the multidrug resistance-associated protein 3 and 4 (MRP3, MRP4) transporters eliminate bile acid from the hepatocyte into the blood in order to control intracellular bile acid accumulation. Moreover, the multidrug resistance-associated protein 2 (MRP2) can eliminate divalent bile acids from the hepatocyte [82]. Therefore, in the screening of drug compounds with toxic potential, it is more accurate to consider BSEP inhibition along with the activity of other liver transporters [83,84].

### 4.2. Host Factors

#### 4.2.1. Age

Aging is known to cause pharmacokinetic changes due to decreased renal function, hepatic mass, blood flow and cytochrome-mediated hepatic metabolism. Furthermore, age-related lean body mass reductions can affect the volume of distribution [85]. Despite these changes liver function is essentially preserved in healthy older humans. However, additional cellular stress factors may contribute to reach a threshold for DILI development in susceptible patients. Older age, as a DILI risk factor is, in fact, a long-term assumption that has not been supported by data from large national DILI registries. In the Spanish DILI Registry 46% of DILI patients were ≥60 years old at the time of the episode and the US Drug-Induced Liver Injury Network (DILIN) reported 16.6% of DILI patients to be 65 years or older [6,27]. The Iceland population-based study [5] observed a relationship between the DILI incidence and the age increase that paralleled to the growing number of prescribed drugs in elderly people. Nevertheless, age appears to be particularly important in specific forms of DILI. For example, children under the age of ten have a higher risk of developing valproic acid-induced hepatotoxicity, with the risk of fatal outcomes being maximal in children below the age of two, possible due to differences in drug metabolism and reduced plasma protein binding [86]. In contrast, the risk of hepatotoxicity induced by isoniazid appears to increase linearly with age, being almost five times higher in patients older than 50 as compared with young patients [87]. On the other hand, the association of Reye syndrome with acetylsalicylic acid is only seen in children [88]. Advancing age has been shown to influence the DILI phenotype; the cholestatic type being more common in older than 60 years [27].

#### 4.2.2. Gender

For some drugs like diclofenac, tetracyclines and nitrofurantoin, liver damage occurs more frecuently in females [89,90]; but cohort studies have failed to demonstrate differences in gender distribution when all patients are considered [2,5,6]. The largest prospective cohorts from Spain with over 600 cases [27] and USA with 900 subjects had 49% and 59% females respectively. Female sex influenced the phenotype in both registries as more hepatocellular injury was seen in women compared with males [2,6,27]. Besides this, female sex was an independent risk factor for fulminant outcome in DILI in the Spanish Registry; 89% of ALF patients were females [2]. In a large cohort of the Acute Liver Failure Study Group (*n* = 133), 77% of cases were women [10].

#### 4.2.3. Race

Ethnical differences in DILI incidence rates among countries can be a reflection of genetic differential background as well as variations in many other factors including life-style, consumption of herbals, regional medication policies and prescription habits. In a multiracial country such as USA, the DILIN group was unable to find an ethnic difference in DILI frequency. However, Asian race was an independent risk factor for transplantation and Afroamerican race was a major determinant for chronic evolution in a 6-months prospective follow-up of the DILIN Registry [91].

#### 4.2.4. Underlying Liver Disease

Pre-existing liver disease is not associated with increased risk of idiosyncratic hepatotoxicity for the bulk of medications, an exception being patients with alcoholic liver disease treated with methotrexate, and patients with chronic B and C hepatitis, especially if they are co-infected with HIV [8]. For example, severe hepatotoxicity caused by antiretroviral medications that contain protease inhibitors, such as ritonavir, is more common among patients co-infected with hepatitis B (HBV) and/or C (HCV) virus, particularly in those not responding to the antiretroviral therapy [92]. Similar predispositions have patients with HBV and/or HCV co-infection when receive non-nucleoside reverse transcriptase inhibitor hepatotoxicity [93,94]. Nevirapine plus a protease inhibitor in patients with underlying HCV infection increased the risk of hepatotoxicity 2.8 times in this last study [94].

Contradicting results with regards to the role of chronic viral co-infections as risk factors in anti-TBC hepatotoxicity have been reported with studies being unable to find any association [95,96] and other demonstrating an increased risk in patients with HBV and HCV co-infection [97,98] up-to14-fold when patients were co-infected with both HIV and HCV [99]. Furthermore, viral load at the time of anti-TBC treatment initiation was found to be a risk factor for DILI development and severity [97]. The mechanism of increased risk of DILI in the setting of chronic viral infections is unclear but virus could act as some form of danger and an altered cytokine milieu as a result of chronic viral diseases could have an effect on hepatic immunity and subsequently contribute towards a break down in immune tolerance in conjunction with drug-induced cellular stress [52]. Besides, individuals with pre-existing liver disease, which mainly included hepatitis C and NAFLD, were found to have higher mortality from DILI (16% *versus* 5.2%) in a recent update of the DILIN database [6]. However, liver-related mortality was not increased in patients with underlying liver disease, indicating that other comorbidities such as diabetes or metabolic syndrome that were more prevalent in that population, may have contributed to the excess of mortality [100].

#### 4.2.5. Comorbidities

An increased risk of toxicity of methotrexate in patients with psoriasis was observed compared to patients with rheumatoid arthritis, although there are confounding factors that limit these observations (age, obesity, diabetes mellitus and use of potentially hepatotoxic drugs) [101,102]. Diabetes does not appear to increase the risk of hepatotoxicity, but has been associated with an increased risk of mortality in the DILIN study [6,103] and of chronicity in the Spanish DILI Registry [104].

#### 4.2.6. Drug-Drug Interactions

Drugs and other xenobiotics may theoretically modulate the hepatotoxic potential of other drugs by inducing/inhibiting its metabolism through cytochrome P-450 (CYP) or competing at the level of membrane transporters [105]. The best known example is the negative effect of chronic alcohol consumption (an inducer of CYP2E1) in acetaminophen toxicity [52]. However, for the bulk of idiosyncratic DILI reactions the evidence of drug-drug interactions playing a role is lacking.

A retrospective study from the General Practice Research Database (GPRD) [89] found increased risk of DILI with combinations of two or more hepatotoxic drugs, but this finding has not been so far reported.

In an analysis of patients with statin hepatotoxicity reported to the Swedish Adverse Drug Reactions Advisory Committee, no clinically significant interaction was found with concomitant medication [106].

In summary, Clinical Networks in Drug-Induced Liver Injury have offered an opportunity for advancing Safety Science and translational research in DILI, providing new insights in clinical phenotypes and severity, and allowing performing pharmacogenetic and mechanistic studies. The complex and multilayered aspects of DILI will require in the near future an integrative approach taking into consideration the combined role of genetic and drug-host-enviromental interactions. Systems biology applied to this field is promising and offers the opportunity to identify clinical risk modifiers and improve our comprehension of the multiple factors involved in idiosyncratic DILI and their interaction. Hopefully, this will enable personalized therapy and safer treatment strategies [107]. In the meantime, a better knowledge of the potential for hepatotoxicity of drugs and dietary supplements, as well as liver test monitoring, in cases in which the hepatotoxicity typically occurs on a background of many more cases with minor increases in transaminases could prevent in some instances further damage and severe outcomes. However, this strategy has proven only be of value with isoniazid and other antituberculosis agents [108] because of its unique profile of efficacy and the lack of safer alternatives. Hence, for the bulk of drugs routine liver test monitoring for preventing idiosyncratic DILI is unpractical and can not be currently recommended.

## Figures and Tables

**Table 1:** Drug-induced liver injury (DILI) pattern of damage.

*R* = (ALT Patient/ULN)/(ALP Patient/ULN)	
Hepatocellular	*R* ≥ 5
Cholestatic	*R* ≤ 2
Mixed	*R* > 2 and <5

**Table 2:** Severity of DILI.

Mild	Elevated ALT or ALP Values Reaching Criteria for DILI, but TB < 2 × ULN
Moderate	Elevated ALT/ALP values reaching criteria for DILI and TB ≥ 2 × ULN, or symptomatic hepatitis
Severe	Elevated ALT/ALP values reaching criteria for DILI, T ≥ 2 × ULN, and one of the following: (1) International normalized ratio (INR) ≥ 1.5, (2) ascites and/or encephalopathy, disease duration <26 weeks, and absence of underlying cirrhosis or (3) other organ failure considered to be due to DILI
Fatal	Death or transplantation due to DILI

**Table 3:** Drugs and compounds predominantly associated with hepatocellular, cholestatic or mixed damage. (HC: Hepatocellular, Chol: Cholestatic, Mx: Mixed).

Drug	Type of Liver Damage	Time to Onset	Immunoallergic Features (Rash. Fever, Esonophilia)	Cases of Acute Liver Failure	Cases of Chronic Liver Injury
Acarbose	HC	2–8 months	Not typical	No	No
Albendazole	HC, Mx	Few days–2 months	Maybe present	No	No
Allopurinol	HC, Mx	2–6 weeks	Yes (DRESS syndrome)	Yes	Yes
Amiodarone	HC	Few days–several years	Cases of Reye syndrome	Yes	Yes
Amitryptiline	HC, Chol	1–14 months	Frequent	Yes	Yes
Amoxicillin	HC, Chol	Few days–2 weeks	Yes (Stevens-Johnson syndrome)	Yes	Yes (rare)
Amoxicillin-clavulanic acid	Chol	Few days–8 weeks After antibiotic is completed (few days–6 weeks)	Not prominent	Yes	Yes (rare)
Ampicillin	HC, Chol	Few days–2 weeks	Yes (Stevens-Johnson syndrome)	Yes	Yes
Androgenic steroids	Chol	1–4 months	No	No	No
Asparaginase	HC	2–3 weeks	Rare	Yes	No
Atorvastatin	Chol, Mx, HC	1 month-several years	Yes (autoimmune hepatitis)	Yes	Yes
Azathioprine	Chol	2–12 months	Uncommon	Yes	No
Bupropion	Chol, HC	1–3 months	Uncommon	Yes	No
Captopril	Chol	2–12 weeks	Infrequent	Yes	Yes
Carbamazepine	Mx, Chol, HC	1–8 weeks	Yes (DRESS syndrome)	Yes	Yes
Celecoxib	Chol, HC	Few days-few weeks	Yes (Stevens-Johnson syndrome)	No	Yes
Chlorpromazine	Chol	1–5 weeks	Some cases (mild)	Yes	Yes
Chlorpropamide	Chol, HC, Mx	2–12 weeks	Yes	No	No
Ciprofloxacin	Chol, HC	2 days–2 weeks	Many cases	Yes	No
Clarithromycin	Chol, HC	1–3 weeks	No	Yes (HC cases)	Yes
Clindamycin	HC, Mx	1–3 weeks	Typical	No	Yes
Clopidogrel	HC	2–24 weeks	Mild, not prominent	Yes	No
Cloxacillin	Chol	1–6 weeks	No	No	No
Contraceptives	Chol	Few cycles	No	No	No
Cyproheptadine	Chol, Mx	1–6 weeks	No	No	No
Diazepam	Chol, Mx	1–6 months	No	No	No
Diclofenac	HC	2–6 months	Yes	Yes	Yes (rare)
Dicloxacillin	Chol	1–6 weeks	Yes, not prominent	No	No
Didanosine	HC	Few weeks	Not prominent	Yes	Yes
Disulfiram	HC	2–12 weeks	Not uncommon	Yes	No
Enalapril	Chol	2–12 weeks	Infrequent	Yes	Yes
Erythromycin	Chol	1–3 weeks	Common	Yes	Yes
Fluoxetine	HC	2–12 weeks	No	No	No
Flutamide	HC	1–10 months	Rare	Yes	No
Fluvastatin	Chol, Mx	1–4 months	Uncommon	Yes (rare)	No
Fosinopril	Chol	2–12 weeks	Infrequent	No	Yes
Glibenclamide	Chol, Mx	3–12 weeks	Not typical	Yes	Yes
Gold preparations (iv)	Chol	1–8 weeks	No	Yes	No
Halothane	HC	2–14 days	Yes	Yes	Yes (if repeated exposure)
Ibuprofen	HC, Chol	Few days–3 weeks	Prominent (Stevens-Johnson syndrome)	Yes	Yes
Imipramine	Chol, HC	1–8 weeks	Not prominent	Yes (rare)	Yes (rare)
Indomethacin	HC	1–8 weeks	Not common	Yes (rare)	No
Irbesartan	HC	1–4 weeks	No	No	No
Isoniazid	HC	2 weeks–6 months	Uncommon (mild)	Yes	Yes (rare)
Ketoconazole	HC, Chol	1–6 months	Rare	Yes	Yes (rare)
Leflunomide	Chol, HC	1–6 months	Not prominent	Yes	No
Lovastatin	Chol	Few weeks–several years	No	Yes (rare)	Yes
Mebendazole	HC	Few days	Typical	No	No
Mesalazine	Chol, HC	1–6 months	No	No	No
Methimazole	Chol, Mx	2–12 weeks	Uncommon	Rare	Yes (rare)
Methotrexate		5–10 years	No	No	Yes (cases of cirrhosis)
Minocycline	HC	1–3 months	Common (autoimmune markers)	Yes	Yes
Mirtazapine	HC	Several months–several years	Uncommon	No	No
Nitrofurantoin	HC	1–2 weeks	Typically	Yes	Yes (autoimmune hepatitis)
Nefazodone	HC	6 weeks–8 months	Uncommon	Yes	No
Norfloxacin	HC, Chol	1 day–3 weeks	Many cases	Yes	No
Omeprazole	HC	1–4 weeks	Rare	Yes (rare)	No
Paroxetine	HC, Mx	2–16 weeks	Uncommon	Yes	No
Penicillamine	Chol	1–6 weeks	Common	Yes	Yes
Pentamidine	HC	Few days	No	No	No
Phenytoin	HC	2–8 weeks	Common (DRESS)	Yes	Rare
Pioglitazone	HC, Chol	1–6 months	Rare	Yes (HC cases)	No
Pravastatin	Chol, HC	2–9 months	Uncommon	No	No
Pyrazinamide	HC	4–8 weeks	Uncommon	Yes	No
Risperidone	Chol	Few days (even years)	Rare	No	No
Rofecoxib	Chol, Mx	1–12 weeks	Uncommon	No	No
Rosiglitazone	HC, Chol	1–12 weeks	Rare	Yes (HC cases)	No
Simvastatin	HC, Chol	1–6 months	Uncommon	Yes (rare)	No
Sulfasalazine	Mx	Few days–weeks	Common (DRESS)	Yes	Yes
Sulindac	HC, Mx	Few days–weeks	Prominent	Yes	Yes
Tamoxifen	Chol, Mx, HC	6 months	Uncommon	Yes	Yes (cases of fatty liver)
Telithromycin	HC	Few days–1 week	Uncommon	Yes	No
Terbinafine	HC, Chol	6 weeks	Uncommon (Stevens-Johnson)	Yes	Yes
Thiabendazole	Chol	1–2 weeks	Rare	Yes	Yes
Ticlopidine	Chol	6 weeks	Not common (mild)	Yes	Yes
Tetracycline	HC	Few days	No	Yes (pregnancy)	No
Tolcapone	HC	1–5 months	No	Yes	No
Trazodone	HC	Few days–6 months	Not prominent	Yes (rare)	Yes (rare)
Trimethoprin-sulfamethoxazol	Chol, Mx	Few days–weeks	Common (DRESS syndrome)	Yes	Yes
Troglitazone	HC	1–6 months	Uncommon	Yes	Yes
Valproic acid	HC	1–6 months	Rare	Yes (Reye like-syndrome)	Yes (cases of cirrhosis)
Venlafaxine	Chol, HC	1–3 months	Uncommon	No	No
Verapamil	Mx, Chol	2–8 weeks	Rare	No	No
Zidovudine	Chol	1–4 weeks	Not common	Yes	No

**Table 4:** Clinicopathological Patterns of DILI.

Type of Damage	Drug	Histological Features
Acute hepatocellular injury	Isoniazid, aspirin, sulfamides	Lobular predominant lymphocytic-plasmacytic infiltration +/− hepatocellular degeneration, lobular disarray, no cholestasis
Autoimmune-like hepatitis	Nitrofurantoin, minocycline, Ipilimumab	Plasma cells and interface hepatitis
Pure cholestasis	Anabolic steroids, estrogens	Hepatocyte cholestasis and dilated biliary canaliculi with bile plugs, without evidence of necrosis or inflammation
Cholestasis hepatitis	Phenytoin, amoxicillin-clavulanate, fluorquinolones, macrolides, azithromycin	Portal and ductal inflammation as well as hepatocyte necrosis with marked predominance centrilobular cholestasis
Granulomatous hepatitis	Isoniazid, interferon, phenytoin, allopurinol	Nonnecrotizing epithelioid granulomas
Chronic hepatitis	Diclofenac, Methyldopa, Bentazepam	Portal predominant, interface hepatitis, portal-based fibrosis
Macrovesicular steatosis	Tetracycline, steroids, gold, 5-fluorouracil, methotrexate, tamoxifen	Variable degrees of accumulation of large fat droplets with peripheral displacement of the nucleus without significant inflammation or cholestasis or alternate pattern
Microvesicular steatosis	Valproic acid, tetracycline, zidovudine	Diffuse hepatocyte accumulation of small fat droplets maintaining a central placement of the nucleus without significant inflammation or cholestasis or alternate pattern
Non-alcoholic fatty liver	Tamoxifen, amiodarone	Macrosteatosis and microsteatosis, hepatocyte ballooning and periportal inflammation
Vanishing bile duct syndrome	Amoxicillin-clavulanate, sulfonamides	Paucity of interlobular bile ducts
Fibrosis/cirrhosis	Methotrexate, amiodarone	Hepatic collagenization with minimal inflammation
Sinusoidal obstruction syndrome	Busulfan, oxaliplatin	Sinusoidal dilatation and congestion, central venule occlusions, perisinusoidal fibrosis
Liver adenoma	Oral contraceptives	Normal appearance of the hepatocytes. These are arranged in sheets and have no malignant features. These cells tend to be larger than normal hepatocytes, and their cytoplasm often contains fat or glycogen

**Table 5:** HLA alelles associated to hepatotoxicity.

Compounds	Number of Cases	HLA Allele	Odds Ratio (95% CI)	*p* Value
Flucloxacillin	51	B*57:01	80.6 (22.8–284.9)	9 × 10^−19^
Amoxicillin-clavulanate	201	A*02:01	2.3 (1.8–2.9)	1.8 × 10^−10^
		DRB1*15:01-DQB1*06:02	2.8 (2.1–3.8)	3.5 × 10^−11^
Lumiracoxib	41	DRB1*15:01-DQB1*06:02	5.0 (3.6–7.0)	6.8 × 10^−25^
Lapatinib	35	DRB1*07:01-DQA1*02:01	2.9 (1.3–6.6)	0.007
Ximegalatran	74	DRB1*07:01-DQA1*02:01	4.4 (2.2–8.9)	6 × 10^−6^
Ticlopidine	22	A*33:03	13.0 (4.4–38.6)	1.2 × 10^−5^
Terbinafine	14	A*33:01	40.53 (12.51–288.9)	6.7 × 10^−10^
Fenofibrate	7	A*33:01	58.7 (12.31–279.8)	3.2 × 10^−7^
Ticlopidine	5	A*33:01	163.1 (16.2–1642)	0.00002

## Abbreviations

DILI	Drug-Induced Liver Injury
GWAs	Genome-wide association study
HDS	Herbal and Diet Supplements
ALF	Acute liver failure
DILIN	Drug Induced Liver Injury Network
ALT	Alanin-aminotransferase
ULN	Upper limit of normal
ALP	Alkaline Phosphatase
TB	Total Bilirrubin
AST	Aspartate-aminotransferase
OLT	Orthotopic liver transplantation
GGT	G-glutamyl transpeptidase
AIH	Autoimmune hepatitis
RUCAM	Roussel Uclaf Causality Assessment Method
CTLA-4	Cytotoxic-T lymphocyte A-4
PD-1	Programed death-1
SOS	Sinusoidal obstruction syndrome
HLA	Human Leukocyte Antigen
DAMPS	Damage Associated Molecular Patterns
FDA	Food and Drug Administration
ROS	Reactive oxygen species
mtDNA	Mitochondrial deoxyribonucleic acid
BSEP	Bile salt export pump
MRP	Multidrug resistance-associated protein
HIV	Human immunodeficiency virus
HBV	Hepatitis B virus
HCV	Hepatitis C virus
NAFLD	Non-alcoholic fatty liver disease
CYP	Cytochrome
GPRD	General Practice Research Database
